# Supplementary material for: Single cell atlas of Xenoturbella bocki highlights limited cell-type complexity
Source: Nat Commun. 2024 Mar 19;15:2469. doi: 10.1038/s41467-024-45956-y (PMC10951248; doi:10.1038/s41467-024-45956-y)
Supplement: Supplementary file 1 — Supplementary Information [file 41467_2024_45956_MOESM1_ESM.pdf]

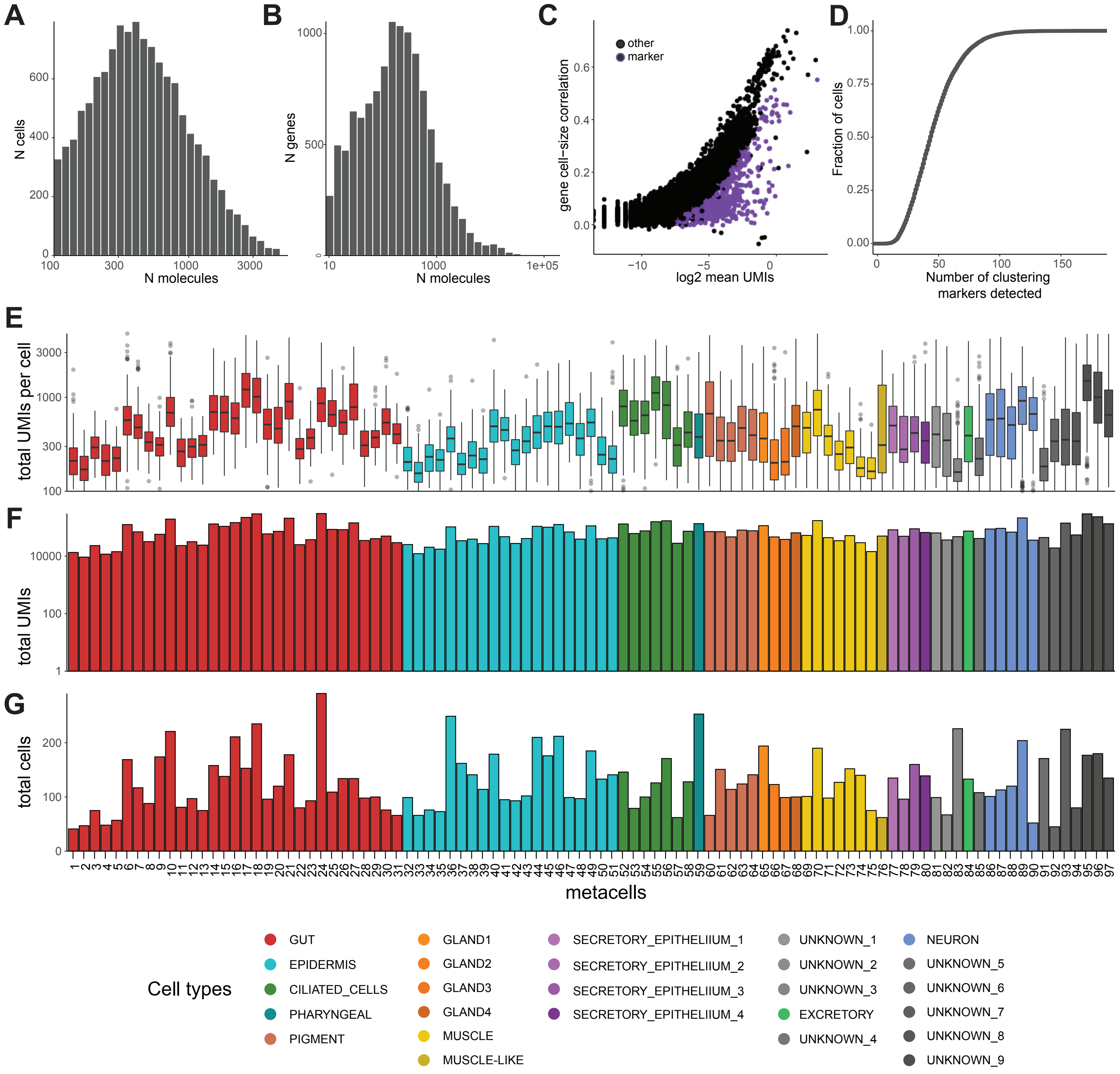

Figure S1. ***Xenoturbella*** **scRNA-seq atlas statistics.**
a Distribution of total RNA molecules per cell. **b** Distribution of total RNA molecules per gene. **c** Relationship between gene total expression (x axis) and the correlation between gene expression and total RNA molecules per cell (y axis). Marker genes selected for cell clustering are shown in purple. **d** Cumulative distribution of number of marker genes detected per single cell. **e** Distribution of RNA molecules per cell per metacell. **f** Total number of RNA molecules per metacell. **g** Number of cells per metacell.

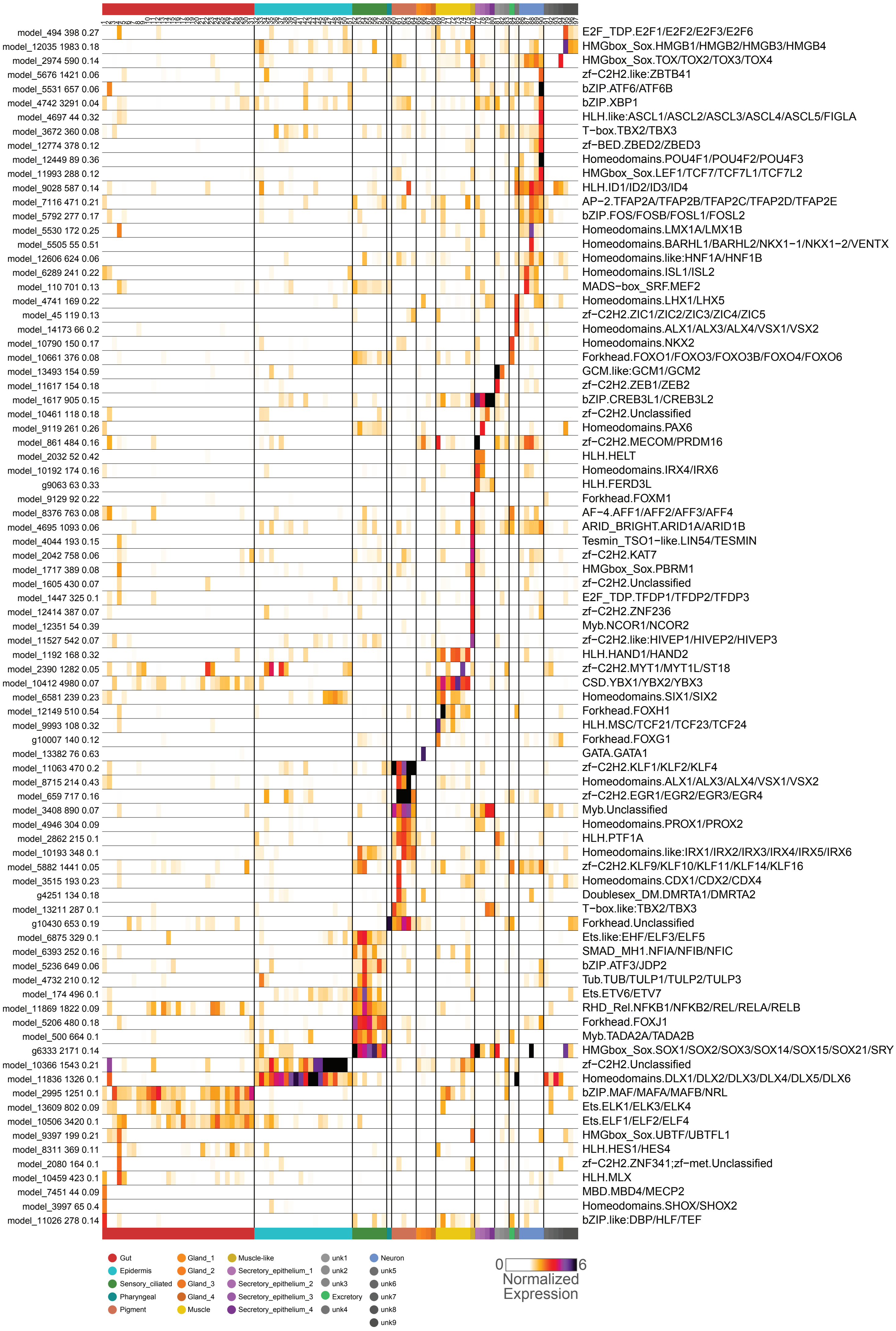

Figure S2. Transcription factor expression profile across *Xenoturbella* metacells. On the right side, the structural class and the phylogeny-based family classification for each TF is indicated. Cell type annotations are colour-coded above/below the heatmap.

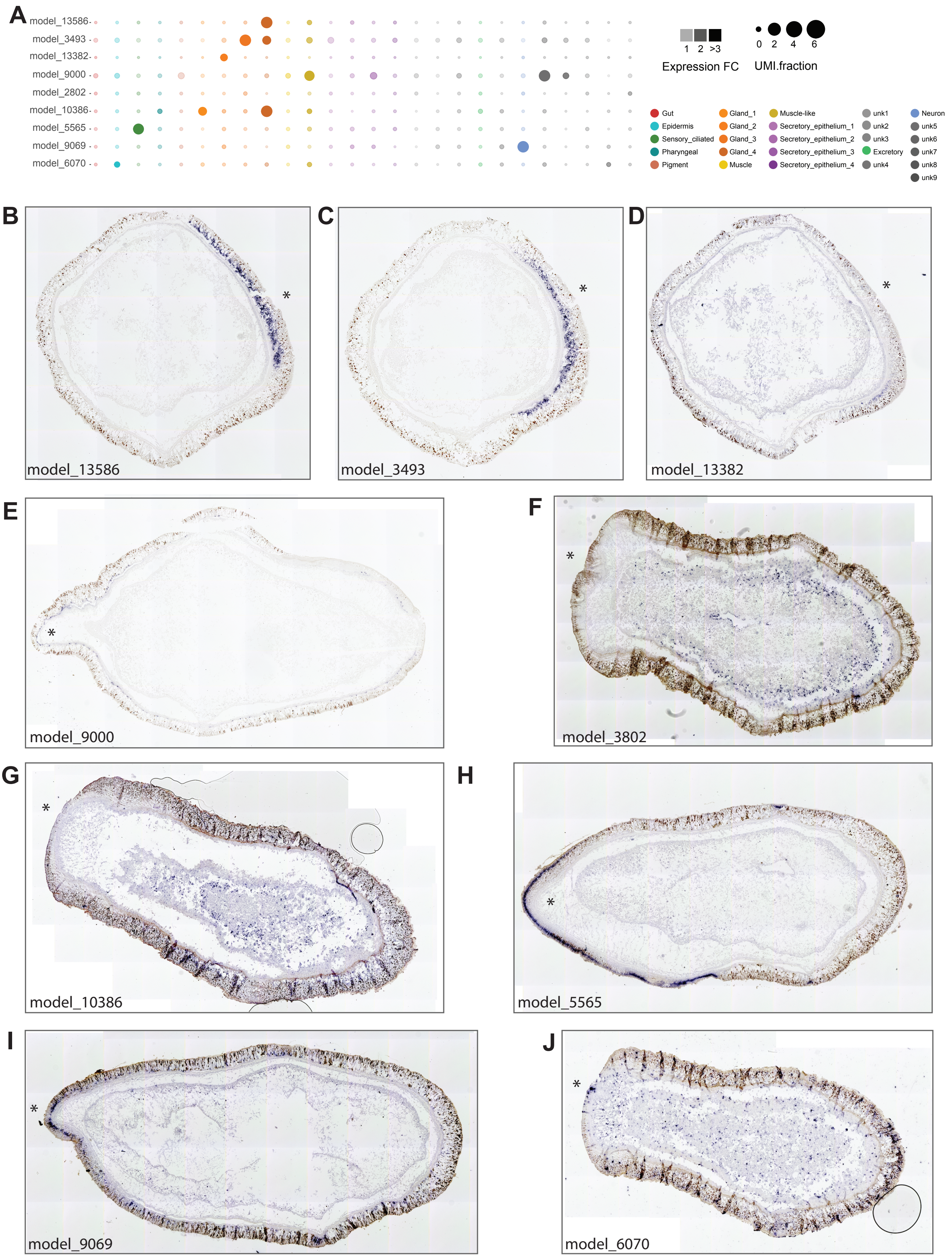

Figure S3. Additional markers validated by ISH.
a Dot plot of additional genes included for ISH across metacells. **b** ISH for Xb model_13586, expression localised to the basal portion of the epidermis in the anterior of the animal. In all panels, the anterior of the animals is indicated by *. **c** ISH for Xb model_3493, expression localised to the basal portion of the epidermis in the anterior of the animal. **d** ISH for Xb model_13382 (GATA3), expression localised to a specific patch of cells in the anterior epidermis. Probes for **b**, **c** and **d** all correspond to genes specifically expressed in three different gland populations. **e** ISH for Xb model_9000 localising to specific cells in the apical portion of the muscle. **f** ISH for Xb model_3802, localising to the gut. **g** ISH for Xb model_10386, localising to digestive gland cells. **h** ISH for Xb model_5565,, expression in the ciliary furrow and the nerve plexus in the anterior region of the section. **i** ISH for Xb model_9069 (adenosine receptor), expression in nerve plexus in anterior of section. **j** ISH for Xb model_6070, broadly expressed across the gut and in the epidermis

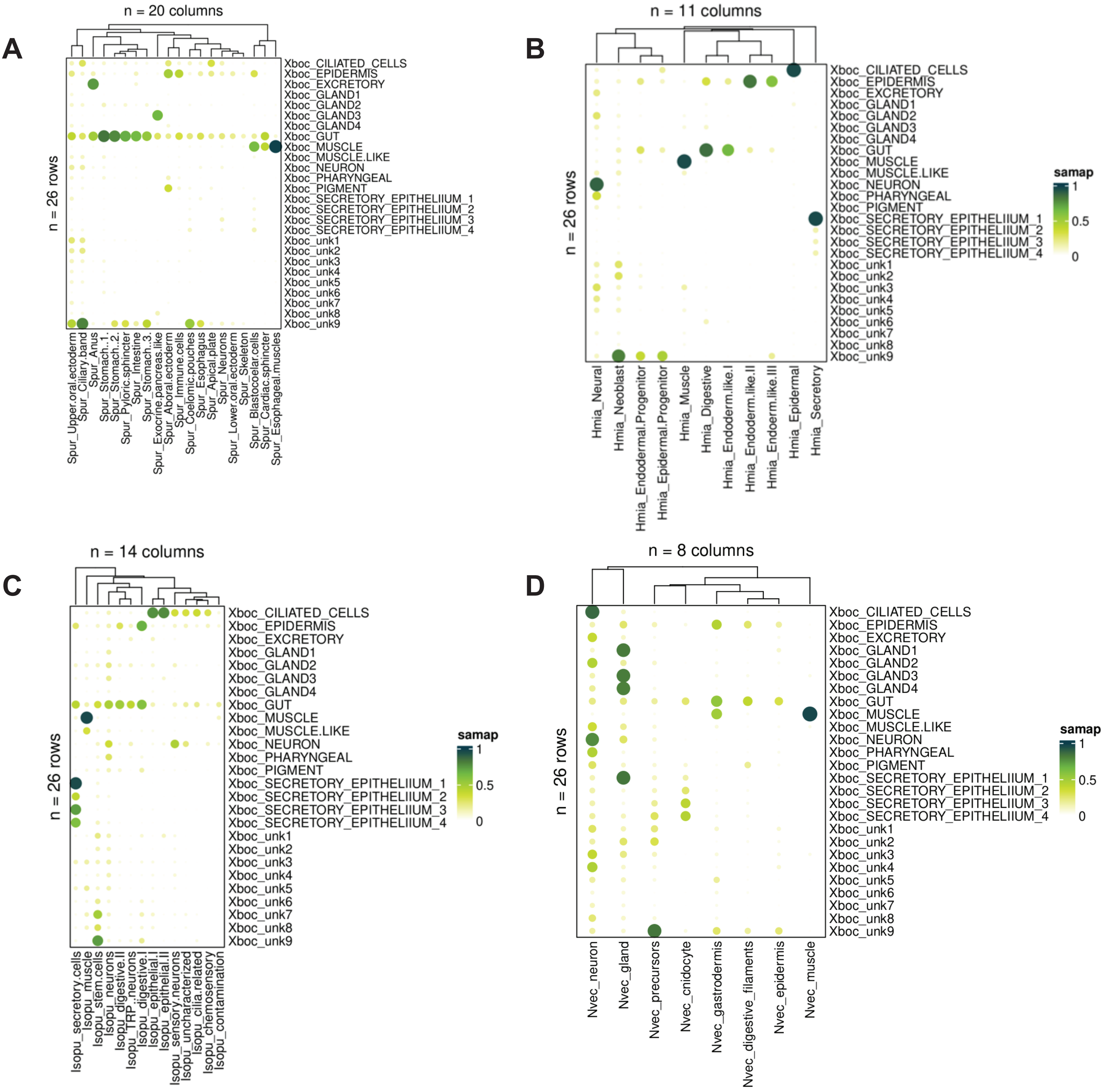

Figure S4. Cross-Species Analysis of Cell Cluster Similarity.
SAMap similarity analysis was performed according to [50] pairwise on the *Xenoturbella* scRNA-seq data and publicly available data [30,31,37,51] from **a** sea urchin **b** *H. miamia* (acoel) **c** *I. pulchra* (acoel) and **d** *N. vectensis* (sea anemone).

Batch	Specimen	Nreads	Nmolecules	Median_reads_per_cell	Perc_mapping	10_percentile_umis	50_percentile_umis	90_percentile_umis	N_cells_over100umis
a170424_XbA1_P01A	Specimen_1	9894088	153863	28358	27.93	136	458	1865	184
a170424_XbA1_P01H	Specimen_1	13730108	203218	37713	25.81	154	650	2447	187
a170424_XbA1_P02A	Specimen_1	22500828	101164	61624	15.72	140	351	1065	181
a170424_XbA1_P02H	Specimen_1	22144325	129182	49120.5	17.61	181	432	1405	188
a170424_XbA1_P03A	Specimen_1	21224874	126071	58959	24.15	106	372	1651	179
a170424_XbA1_P03H	Specimen_1	15472882	100117	50619.5	24.42	168	430	970	187
a170424_XbA1_P04A	Specimen_1	40950905	230287	106276.5	26.58	365	852	2164	191
a170424_XbA1_P04H	Specimen_1	16184610	123428	39094.5	29.14	137	348	1529	179
a170424_XbA1_P05A	Specimen_1	12529655	116387	43000.5	25.23	102	430	1291	173
a170424_XbA1_P05H	Specimen_1	9408009	111364	26592	22.18	92	362	1340	172
a170424_XbA1_P06A	Specimen_1	13722265	163154	43208	25.15	188	598	1621	182
a170424_XbA1_P06H	Specimen_1	9158398	129653	23047	25.58	103	352	1729	174
a170424_XbA1_P07A	Specimen_1	10649461	126825	27714.5	23.45	126	382	1534	180
a170424_XbA1_P07H	Specimen_1	11965341	130081	35948.5	22.01	133	430	1579	182
a170424_XbA1_P08A	Specimen_1	10222890	138738	29288.5	24.96	131	412	1750	178
a170424_XbA1_P08H	Specimen_1	13884937	110333	36650.5	23.02	99	366	1148	171
a170424_XbA1_P09A	Specimen_1	8906340	112226	26790.5	24.14	182	414	1337	184
a170424_XbA1_P09H	Specimen_1	7346216	112143	21148	21.71	112	390	1206	176
a170424_XbA1_P10A	Specimen_1	10172198	147371	28662	24.9	195	554	1650	190
a170424_XbA1_P10H	Specimen_1	9211564	122133	24445.5	22.49	131	439	1389	184
a170424_XbA1_P11A	Specimen_1	8215636	111502	25242.5	23.09	154	382	1166	186
a170424_XbA1_P11H	Specimen_1	5791725	68559	15620	20.53	77	233	847	160
a170424_XbA1_P12A	Specimen_1	8806539	141467	28497	22.76	212	495	1506	189
a170424_XbA1_P12H	Specimen_1	8165938	117840	24157.5	18.79	159	484	1371	181
a170424_XbA1_P13A	Specimen_1	9180312	110181	20721.5	21.47	117	325	1425	177
a170424_XbA1_P13H	Specimen_1	8042273	92643	22302	21.72	89	369	998	171
a170424_XbA1_P14A	Specimen_1	13757652	131110	48557	19.15	229	557	1283	190
a170424_XbA1_P14H	Specimen_1	15907477	120752	44515	20.7	122	434	1330	183
a170424_XbA1_P15A	Specimen_1	11563243	97776	33607	21.76	113	392	1093	179
a170424_XbA1_P15H	Specimen_1	14950290	97629	34124.5	19.16	113	356	1205	178
a170424_XbA1_P16A	Specimen_1	10020110	68479	30807.5	23.9	97	261	750	171
a170424_XbA1_P16H	Specimen_1	7129941	64959	23773	24.6	81	242	650	162
a171115_Xb_A1_P01A	Specimen_2	18455452	85082	63357	19.79	88	306	776	169
a171115_Xb_A1_P01H	Specimen_2	13489438	101010	49072	20.48	147	424	918	178
a171115_Xb_A1_P02A	Specimen_2	17915618	112134	51791.5	19.84	199	428	939	182
a171115_Xb_A1_P02H	Specimen_2	12145265	58307	42479	20.14	58	236	556	160
a171115_Xb_A1_P03A	Specimen_2	14360748	65837	58157.5	23.02	120	309	604	176
a171115_Xb_A1_P03H	Specimen_2	14802290	76127	49332.5	22.24	92	331	737	171
a171115_Xb_A1_P04A	Specimen_2	13244593	57922	42798	23.67	105	236	515	174
a171115_Xb_A1_P04H	Specimen_2	18543530	73314	72036.5	25.64	110	316	693	175
a171115_Xb_A1_P05A	Specimen_2	17048733	75954	67606	18.77	172	346	707	182
a171115_Xb_A1_P05H	Specimen_2	18498921	57535	69703.5	17.28	70	234	573	166
a171115_Xb_A1_P06A	Specimen_2	8747756	57268	35108.5	22.38	117	260	510	174
a171115_Xb_A1_P06H	Specimen_2	17145155	60775	67289	22.6	83	267	530	167
a171115_Xb_A1_P07A	Specimen_2	17164694	67803	36050	10.64	99	288	667	172
a171115_Xb_A1_P07H	Specimen_2	14324461	60999	47235.5	18.87	68	276	598	161
a171115_Xb_A1_P08A	Specimen_2	14391963	64266	56108	24.74	136	310	557	177
a171115_Xb_A1_P08H	Specimen_2	16238039	66747	61813	25.7	108	280	631	175
a171115_Xb_A1_P09A	Specimen_3	21039895	269545	87805	20.8	290	1132	2960	189
a171115_Xb_A1_P09H	Specimen_3	15716597	197192	60947.5	19.26	183	912	2260	183
aRP0092A_170425_Xb_A3_iX17	Specimen_3	6920821	172162	14499	34.5	172	401	1204	191
aRP0092H_170425_Xb_A3_iX18	Specimen_3	7390181	222766	14566.5	37.59	192	486	1328	190
aRP0093A_170425_Xb_A3_iX19	Specimen_3	5161353	90702	13145.5	32.95	125	296	1090	185
aRP0093H_170425_Xb_A3_iX20	Specimen_3	6464864	115591	17993.5	37.53	122	330	1243	185
aRP0094A_170425_Xb_A3_iX21	Specimen_3	5981757	78204	15451	22.79	118	256	899	182
aRP0094H_170425_Xb_A3_iX22	Specimen_3	5208645	85530	12283	26.8	103	225	1009	175
aRP0095A_170425_Xb_A3_iX23	Specimen_3	6503734	112615	18286	24.12	169	388	1168	191
aRP0095H_170425_Xb_A3_iX24	Specimen_3	6768486	134159	19177	28.2	195	444	1522	191
aRP0096A_170425_Xb_A3_iX25	Specimen_3	5978594	127916	15201	23.12	113	234	1060	179
aRP0096H_170425_Xb_A3_iX26	Specimen_3	7784085	638331	3666	28.75	147	273	890	190
aRP0097A_170425_Xb_A3_iX27	Specimen_3	6542369	102021	17165.5	24.18	109	303	1097	181
aRP0097H_170425_Xb_A3_iX28	Specimen_3	6054683	97336	15599	26.67	108	258	1119	175
aRP0098A_170425_Xb_A3_iX29	Specimen_3	5443345	106601	13023.5	29.01	107	247	1427	176
aRP0098H_170425_Xb_A3_iX30	Specimen_3	6355872	121442	14816.5	29.67	137	368	1384	187
aRP0099A_170425_Xb_A3_iX31	Specimen_3	5792604	112945	15576.5	30.47	112	308	1321	178
aRP0099H_170425_Xb_A3_iX32	Specimen_3	6151549	117541	15309	31.85	129	310	1396	186
aRP0545A_180504_Xb_A5_SpT4_i	Specimen_4	7377256	155622	23181	32.09	160	552	1627	185
aRP0545H_180504_Xb_A5_SpT4_i	Specimen_4	6704298	129863	23125	29.06	203	490	1289	188
aRP0546A_180504_Xb_A5_SpT4_i	Specimen_4	6080463	106701	20588	26.9	129	390	1178	182
aRP0546H_180504_Xb_A5_SpT4_i	Specimen_4	7619165	128028	25338	29.12	172	492	1411	189
aRP0555A_180504_Xb_A5_SpT10_	Specimen_4	7523015	117890	25441	27.18	79	419	1322	169
aRP0555H_180504_Xb_A5_SpT10_	Specimen_4	6851313	125417	24657	27.65	176	500	1358	186
aRP0556A_180504_Xb_A5_SpT10_	Specimen_4	5920447	152087	20973.5	27.9	208	606	1577	189
aRP0556H_180504_Xb_A5_SpT10_	Specimen_4	6260769	125899	24221.5	29.76	125	522	1252	180
aRP0561A_180504_Xb_A5_nST4_i	Specimen_4	7434722	165367	23815	30.29	163	658	1690	187
aRP0561H_180504_Xb_A5_nST4_i	Specimen_4	6077556	145153	19555	28.61	116	460	1661	178
aRP0562A_180504_Xb_A5_nST4_i	Specimen_4	8233399	175883	29654	27.71	269	674	1713	190
aRP0562H_180504_Xb_A5_nST4_i	Specimen_4	4692576	86736	15794.5	27.31	64	313	1023	146
aRP0571A_180504_Xb_A5_nST10_	Specimen_4	6585865	132267	20765.5	29.09	168	476	1325	188
aRP0571H_180504_Xb_A5_nST10_	Specimen_4	6807004	137294	23856.5	28.13	163	462	1376	183
aRP0572A_180504_Xb_A5_nST10_	Specimen_4	6523694	162614	22608	29.75	207	615	1665	188
aRP0572H_180504_Xb_A5_nST10_	Specimen_4	8302002	155294	29133	26.29	167	618	1694	187

Table S1. Metadata associated with each half-plate of single cell RNA-seq samples.

Model	Forward primer	Reverse primer
13733	ACGATTGGTGCCAAGAAAAC	TTAAATGGGAATGGCTCGTC
8649	ATCGCAATCGCAGAATTCAA	TTAGGCCTAGTCCAGTGACACAT
3275	GTACATACGCATCTCCGCTCATC	AATACATTCCAACGGTCCTCCTC
4365	ATAGCTGAGTCGCGCATACACAT	AATTCAGCCGCTCGTTCCTTAG
3304	ACACAGAGACAGCTGGTGATGCT	GCTAACATATCCGACCAATGACG
8066	CGCTGGTAGAACTGGAATACAGC	TGGATGGTCTCTCTTGATTGTCC
179	ACTTCAACACACGACGCAATCTT	GCATATGCACGTGAACACGAA
14024	CGAACGAGTCGGTTGAACAACTA	ATGAACGCGTGATGATGAGTAGG
7170	GAAGGAGTATCACAGCGATGGAA	GGTGCAAGGAACTGATTATGGTG
5206	GACCCAAAGGTCGTTGTCAT	GTTGGAACGCGATCAATTTT
13586	CGAGACATACGGCAGCATTC	CGGAGCGAAACTGACAATGT
13382	ACGCGATCGGTCGATACTAC	GGCGAATTTCAAAGTGTCGT
9000	CAACATCGGGAACAGTGTGC	GTTTGCCCAAGAAACGAAACG
5565	GATCTCGTCCAGGATGAAGGTCT	CCGTGCAAGAGATATGTTCAATG
3802	GGATCTCCTAACCACAATTGCTT	GGTCAACCTCCATCAAGTGCAG
9069	AACTCGGACTCAACGCTTCAACT	GCGTTCAATTGATTCCTCGTAGA
6070	GCAATGGACTACCAATGGACAAC	ACGACATTATCACCGTCACGTCT
3493	TTGTGGTTGTGGTTGGAGAG	ACATTATCAGGGGACGGTGT
10386	TTCACCGTGCACTACTCAACTCA	TGGCCTAATATGAGCGGACTGTA

Table S2. Primer sequences used to amplify DNA templates for RNA *in situ* hybridization experiments
