# Supplementary material for: Single cell atlas of Xenoturbella bocki highlights limited cell-type complexity
Source: Nat Commun. 2024 Mar 19;15:2469. doi: 10.1038/s41467-024-45956-y (PMC10951248; doi:10.1038/s41467-024-45956-y)
Supplement: Supplementary file 3 — Description of Additional Supplementary Files [file 41467_2024_45956_MOESM3_ESM.pdf]

Description of Additional Supplementary Files

File Name: Supplementary Data 1
Description: Xenoturbella_metacell_gene_expression

File Name: Supplementary Data 2
Description: Xenoturbella_metacell_UMI_counts
